# Displaced intra-articular calcaneal fracture with comminuted calcaneocuboid depression fracture: case report

**DOI:** 10.1016/j.tcr.2025.101205

**Published:** 2025-05-26

**Authors:** Motoyuki Takaki, Akira Yoshino, Hirono Ojima, Takuya Yuki, Asami Morimoto, Narutaka Katoh

**Affiliations:** a Department of Traumatology, Fukushima Medical University, 1Hikariga-oka, Fukushima 960-1295, Japan; b Trauma & Reconstruction Center, Southern TOHOKU General Hospital, 7-115 Yatsuyamada, Koriyama 963-8563, Japan

**Keywords:** Calcaneal fracture, Calcaneocuboid joint depression fracture, Reduction, Plate fixation, Sinus tarsi approach

## Abstract

**Background:**

Although it is well known that some calcaneal fractures extend to the calcaneocuboid joint, there have been no reports detailing the techniques of reduction in calcaneal fractures with severe displaced depressed calcaneocuboid joint fractures.

**Purpose:**

We experienced a case of calcaneal fracture with severe displaced comminuted calcaneocuboid joint depression fracture. The purpose of this report is to introduce reduction and fixation techniques.

**Case:**

51-Year-old male. He was injured after falling from a height of 6 m while pruning trees at his workplace. He was carried to his previous hospital and was urgently hospitalized with a diagnosis of left calcaneus comminuted fracture, left ankle lateral malleolar fracture, 1st lumbar compression fracture, and sacral fracture.

The patient presented with a depression type calcaneal fracture including an intraarticular depression fracture of the calcaneocuboid joint. At surgery, the depression fracture of the calcaneocuboid joint was directly molded to the cuboid, before the posterior talocalcaneal fracture was reduced and temporarily fixed to the cuboid bone with a Kirshner wire. The lateral column was opened using a spreader and shortening of the lateral column was reconstructed when the posterior talocalcaneal intra-articular fracture was reduced.

**Discussion:**

In calcaneal fractures involving the calcaneocuboid joint, those with minor displacement are often reduced indirectly by posterior facet reduction. In cases of depression fractures of the calcaneocuboid joint with severe displacement, there is a risk of shortening of the lateral column, so length of the lateral column must be restored sufficiently. We think that dissection of the fracture site and temporary joint fixation is required for reduction.

**Conclusion:**

For calcaneal fractures with intra-articular depression fractures of the calcaneocuboid joint, reduction techniques to reconstruct the length of the lateral column using a lamina spreader are useful, such as temporary joint fixation after reduction of the calcaneocuboid joint using Kirshner wire.

## Introduction

It is well known that some calcaneal fractures extend into the calcaneocuboid joint, with various authors reporting rates of 38–68 % ([1], [2], [3], [4]). Minor displaced intra-articular fractures of the calcaneocuboid joint are often reduced indirectly by posterior facet reduction, but there are no detailed reports describing the method of reduction and fixation of severe displaced comminuted calcaneal fractures with calcaneocuboid joint depression fractures.

## Purpose

We experienced a case of calcaneal fracture with comminuted calcaneocuboid joint depression fracture. The purpose of this report is to introduce our reduction and fixation techniques for the patient.

## Case

**Fig. 1:**
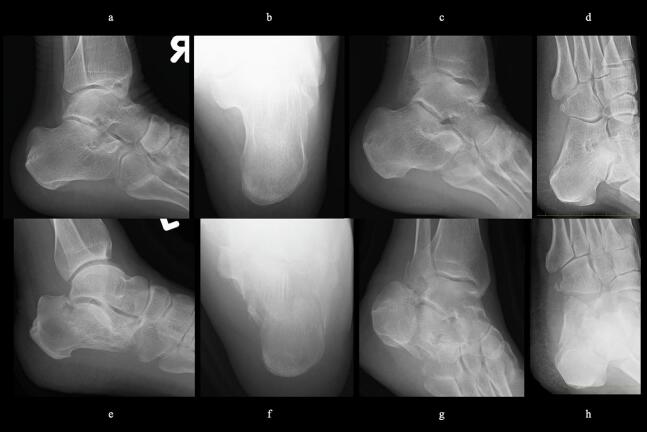
Simple X-ray at the time of injury. a:Lateral view of the affected calcaneus. b:Axial view of the affected calcaneus. c:Broden view on the affected side. d:Oblique view of the affected foot. e:Lateral view of the normal side. f:Axial view of the normal side. g:Broden view of the normal side. h:Oblique view of the normal side.

**Fig. 2:**
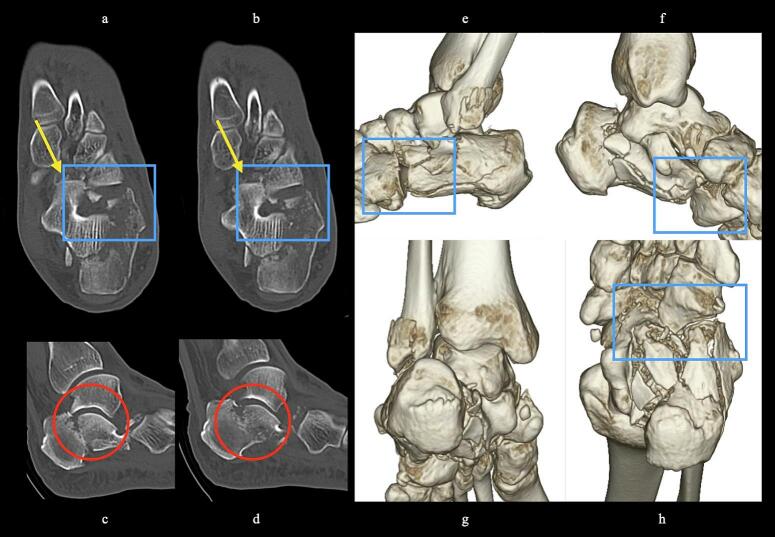
CT at the time of injury. a-b:Horizontal view. Dislocation of the calcaneocuboid joint is present(yellow arrow). Comminuted and depressed fracture of the calcaneocuboid articular surface of the calcaneus(blue box) with marked bulging of the lateral wall. c-d:Siggital view. No comminuting of the posterior talocalcaneal articular surface, but significant depression and the depressed bone fragments are in contact with the basolateral cortex(red circle). Shortening and significant decreesing in calcaneal height are also present. e-h: 3DCT Strong comminuted of the lateral wall and anterior basolateral side, lateral deviation and shortening of the calcaneal tubercle and bulging of the medial and lateral wall are also significant. (For interpretation of the references to colour in this figure legend, the reader is referred to the web version of this article.)

**Fig. 3:**
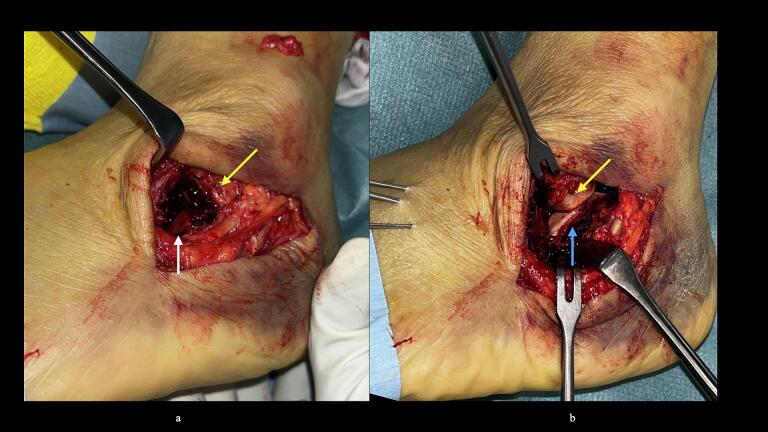
Intraoperative photograph. a:Depressed calcaneocuboid articular surfaces can be identified(white arrow). b:The lateral process of the talus(yellow arrow) and depressed posterior talo-calcaneal articular surface(blue arrow) can be seen. The Kirschner wire visible on the left is a temporary fixation of the repaired calcaneocuboid articular surface. (For interpretation of the references to colour in this figure legend, the reader is referred to the web version of this article.)

**Fig. 4:**
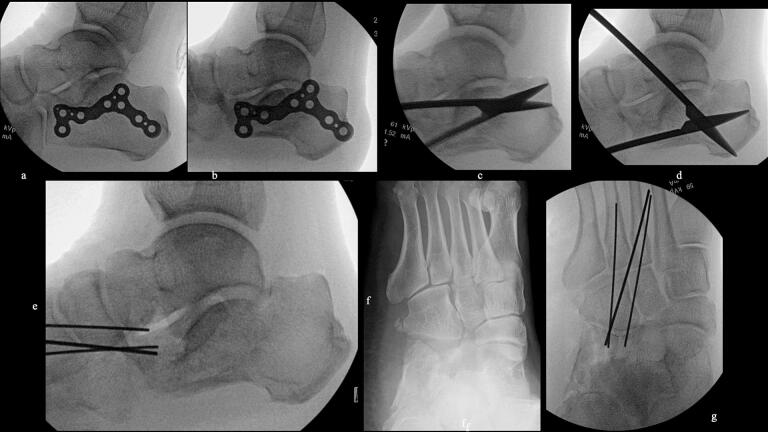
Intraoperative image intensifier. a:Templating of the plate on the normal side. b:Templateing of the plate on the affected side; Comparison with the normal side clearly shows the size of the displacement. c-d:Periosteal release of the plate placement; released using a shear blade. e:Temporary fixation by restoration and rays of light on the cubitus calcanei articular surface. A 1.2 mm Kirschner wire inserted posteriorly was pulled out anteriorly over the foot so that the Kirschner wire would not interfere with the restoration of the shortening of the lateral column. f:Oblique view of the foot before reduction of the depressed fragment of the calcaneocuboid joint of the calcaneus. g:Oblique view of the foot after reduction and temporary fixation of. the depressed fragment of the calcaneocubital joint of the calcaneus.

**Fig. 5:**
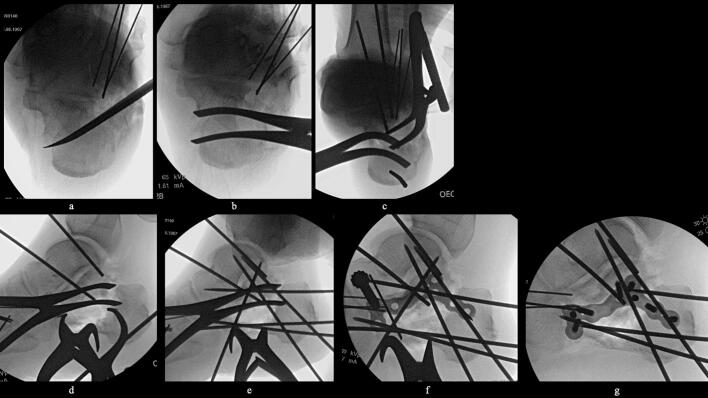
Intraoperative image intensifier. a:Reliese between the posterior talocalcaneal joint depression fragment and the calcaneal tuberosity. b:Openings between the posterior talocalcaneal joint fragment and the calcaneal tuberosity (elevation of the posterior talocalcaneal joint fragment). c:Spreader inserted between the calcaneal base and plantar fascia to open and correct shortening. Posterior talocalcaneal joint depressed fragment elevated and temporarily fixed with 2.4 mm Kirschner wire. d:Gelpy window opener with lateral column lengthning. e:Temporary fixing with Kirschner wire and installation of plates. f:After completion of plate fixation.

**Fig. 6:**
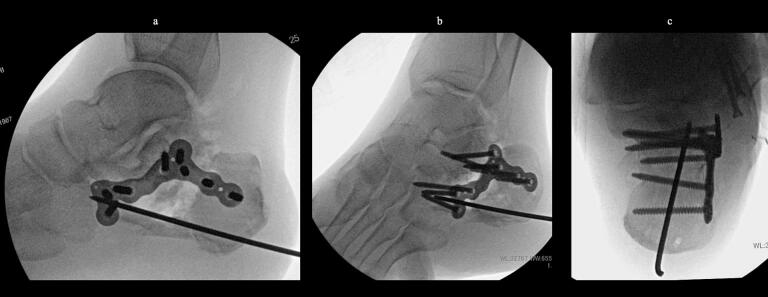
Intraoperative image intensifier. a:Lateral view of the calcaneus after reduction and fixation. b:Broden view after reduction and fixation. c:Axial view of the calcaneus after reduction and fixation.

**Fig. 7:**
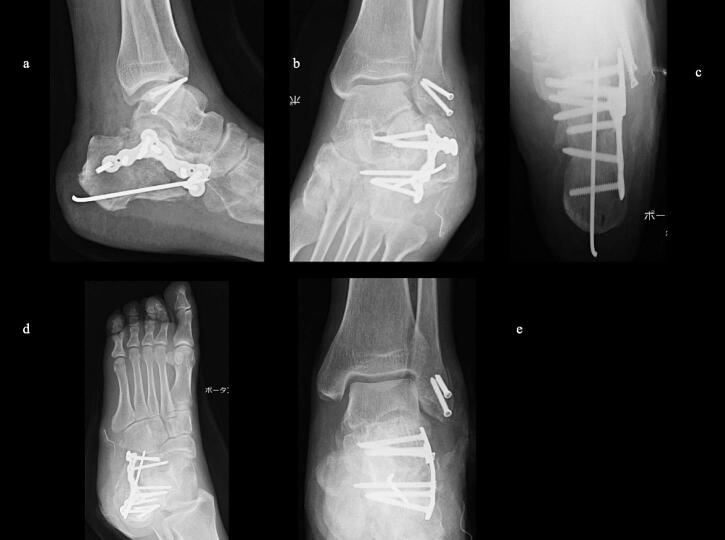
Simple X-ray after operation. a:Lateral view of the calcaneus. Calcaneal height and calcaneal length are reducted. b:Broden view of the calcaneus. Good congruity of the posterior talocalcaneal joint surfaces. c:Axial view of the calcaneus Lateral wall bulging was reduced. d:Oblique view of the foot. Good congruity of the calcaneocuboid articular surface. e:AP view of the ankle The lateral malleolus fracture was fixed with two cortical bone screws.

**Fig. 8:**
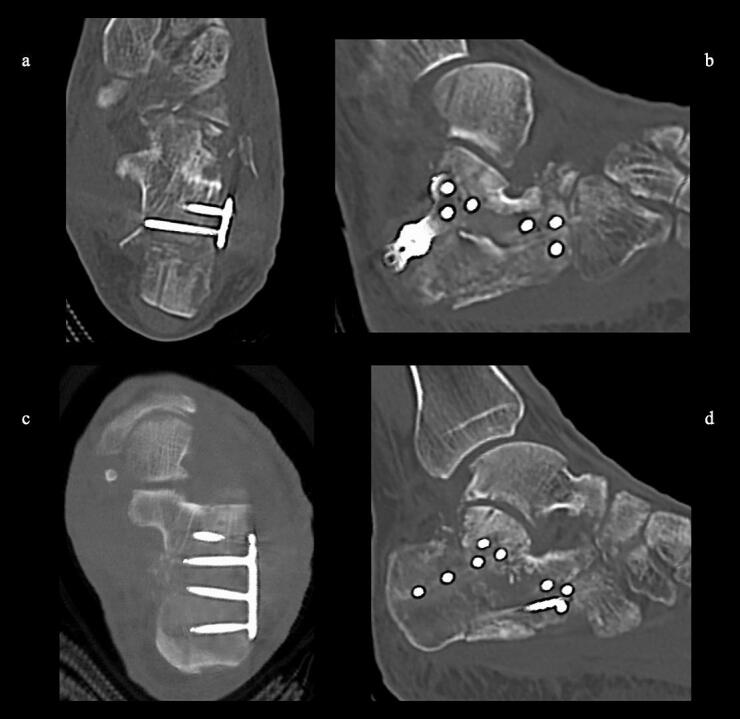
Postoperative 3DCT. a,b: reduction of the calcaneocuboid joint was good. c,d: reduction of the posterior talocalcaneal joint was good.

**Fig. 9:**
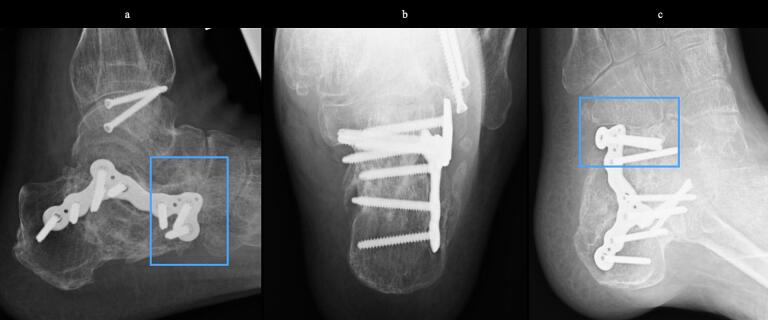
X-ray 7 months after surgery. Bone union without correction loss. a: lateral view of the calcaneus. b: axial view of the calcaneus. c: Oblique view of the foot.

**Fig. 10:**
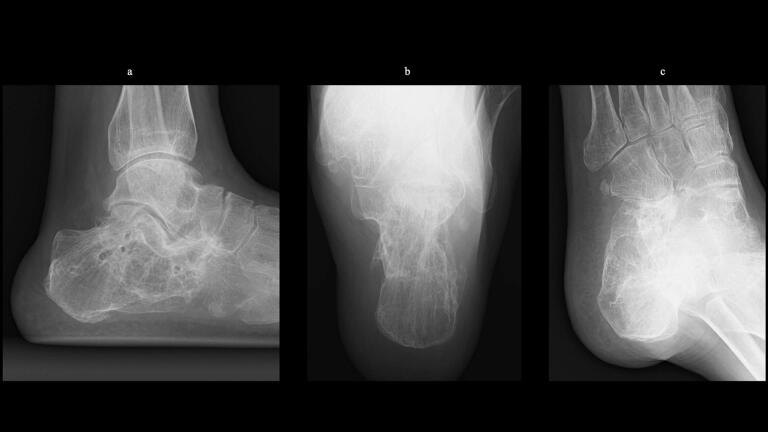
Final X-ray 16 months after surgery. After removal of the plate without correction loss. a: lateral view of the calcaneus. b: axial view of the calcaneus. c: Oblique view of the foot.

51-year-old man. He was injured after falling from a height of 6 m while pruning trees at work. He was carried to his previous doctor and was urgently admitted to hospital with a diagnosis of a left calcaneal comminuted fracture, left ankle lateral malleolus fracture, first lumbar compression fracture and sacral fracture. On 4 days after injury, the patient was transferred to our hospital for surgery. Due to severe swelling of the left foot, Robert Jones bandage immobilisation were started. Lateral view, axial view and Broden view X-ray of the calcaneus, showed a left calcaneal comminuted fracture of depression type, with marked calcaneal shortening, calcaneal height decreesing and calcaneal width widening, with a Böhler angle of 20° compared to 39° on the healthy side (Fig. 1ab). A oblique view X-ray of the left foot showed a depression fracture of the calcaneocuboid articular surface of the calcaneus (Fig. 1d); CT horizontal section showed that the calcaneocuboid articular surface was crushed and the depressed bone fragment was largely divided medially and laterally (Fig. 2ab); 3DCT showed a severe comminution of the calcaneocuboid articular surface and the basolateral anterior aspect (Fig. 2h). The posterior talocalcaneal articular surface was mildly comminuted (Fig. 2cdf). The lateral malleolar fracture was a avulsion fracture of the superior peroneal retinaculum (Fig. 2eg). The lumbar compression fracture, which was only slightly displaced, was treated with orthotics, while the sacral fracture was treated with a conservative treatment. 12 days after injury, when the foot swelling had improved and wrinkles were visible, surgery was performed for the left calcaneal comminuted fracture and the left lateral malleolar fracture. The patient was positioned in lateral decubitus and incised with a sinus tarsi approach with an anterior swing to the base. After identification of the peroneus tendon, a sharp incision was made dorsal to the peroneus tendon anteriorly and basal to the peroneus tendon posteriorly, and the peroneus tendon was elevated with its tendon sheath to expose the lateral calcaneal wall. The lateral aspect of the calcaneal tubercule, which serves as the plate placement area, was also debrided as necessary and sufficient (Fig. 4 cd). The lateral wall was patently dissected and flipped to identify the calcaneocuboid and posterior talocalcaneal articular surface bone fragments (Fig. 3ab). The fracture gap of each fragment and calcaneal tubercule was dissected with a sharp scissors to obtain sufficient mobility of the fragments. The calcaneocuboid articular surface was split medially and laterally, and each bone fragment was conformed to the cuboidal articular surface and temporarily fixed with three 1.2 mm Kirschner wires (Fig. 4eg). A lamina spreader was then applied between the posterior talocalcaneal joint bony fragments and the calcaneal tubercule to spread and attempt to restore calcaneal length and calcaneal height, but a lateral subluxation of the posterior talocalcaneal joint occurred with opening (Fig. 5b). Therefore, the posterior talocalcaneal joint bony fragment was conformed to the talocalcaneal articular surface and temporarily fixed with two 2.4 mm Kirschner wires. When the posterior talocalcaneal joint fragment was again spread between the posterior talocalcaneal joint fragment and the calcaneal tubercule and spread, the calcaneal height was almost as planned and the calcaneal length was also restored to some extent. To reduce the shortening of the remaining calcaneal length, a small medial skin incision was added and a spreader was inserted between the calcaneal base and the plantar fascia and spread (Fig. 5c). To further uniform the shortening at the anterior calcaneus, a Gelpi spreader was opened over the anterior lateral wall of the calcaneus and between the calcaneal tubercule fragments to complete the length of the lateral column (Fig. 5d) A 2.4 mm Kirschner wire was used for temporary fixation and a Biomet MIOS plate was placed (Fig. 5ef). A non locking screw was inserted one into the sustentaculum tali and another into calcaneal tubercule to draw them into the plate and correct in the axial image to achieve a final reduction, and locking screws were inserted as needed to complete the fixation (Fig. 5g). The Kirschner wire of temporary fixation was removed, but considering the severe comminuting of the basolateral cortex, one Kirschner wire on the basolateral side was retained (Fig. 6abc). Postoperatively, calcaneal length, calcaneal height and calcaneal width improved, and the postoperative Böhler angle was 41°, almost equal to that of the healthy side (Fig. 7abc). The calcaneocuboid articular surface was also well reduced (Fig. 7d). Postoperative CT imaging showed good congruency of the calcaneocuboid and subtalar joints, with satisfactory restoration of calcaneal height and length (Fig. 8abcd). Bone union progressed favorably (Fig. 9abc), and implant removal was performed at 12 months postoperatively. At the final follow-up at 16 months, plain radiographs demonstrated maintained joint congruency (Fig. 10abc). The patient had no restriction of range of motion or antalgic gait, and was able to resume jumping and running, returning to his previous occupation. The AOFAS hindfoot scale score was a perfect 100. Outpatient follow-up will be continued to monitor for the development of post-traumatic arthritis.

## Discussion

Among calcaneal fractures, those involving the calcaneocuboid joint have been reported to be relatively common, ranging from 38 to 68 % ([1], [2], [3], [4]) Ebraheim et al. stated that depression fractures of the calcaneocuboid joint are associated with shattering of the lateral column and lateral subluxation of the posterior talocalcaneal joint (1). In the present case, in addition to the comminuted depression of the calcaneocuboid articular surface, there was also significant comminution of the lateral wall and anterior base of the calcaneus. Gallino et al. stated that most calcaneal fractures involving the calcaneocuboid joint have no or mild dislocation and are reduced indirectly by reduction of the posterior talocalcaneal joint (5). In the present case, the dislocation was severe and there was comminution of the joint depression fragments, which required a separate reduction from the posterior talocalcaneal joint. Because of the shattering of the lateral wall, if the depression of the calcaneocuboid joint is not repaired, there is a risk of significant shortening of the lateral column, traumatic arthropathy due to instability, and progress of flatfoot deformity. The technique in this case, which corrects the length, height, width and angle of the calcaneus with temporary joint fixation of the small, unstable fragment, may be very useful in severe comminuted calcaneal fractures. In addition, during the reduction, traction on one of the bone fragments results in a significant loss of force, and opening the space between the bone fragments is the key to the reduction. If the basal fragment is crushed or shortened, there are cases in which the attempt to reduce the bone to a scaffold is not countered and the fragment is instead displaced. In such cases, experience has shown that if a spreader is applied between the basal surface of the calcaneal ridge and the plantaris muscle and the base of the calcaneal ridge is pushed posteriorly, as in this case, length correction is often achieved without causing angular correction loss of the calcaneus or displacement of the basal bone fragments.

## Conclusion

This report describes a method for the reduction of comminuted calcaneal fractures including calcaneocuboid joint depression fractures. Temporary joint fixation of the fracture fragments including the articular surface, spreading between the fragments using a spreader and spreading between the calcaneal base and plantar fascia were very useful for the reconstruction of this case with a depressed calcaneocuboid joint fracture and a shortened lateral column.

## CRediT authorship contribution statement

**Motoyuki Takaki:** Writing – original draft. **Akira Yoshino:** Supervision. **Hirono Ojima:** Validation. **Takuya Yuki:** Validation. **Asami Morimoto:** Validation. **Narutaka Katoh:** Supervision.

## Declaration of competing interest

The authors declare that they have no known competing financial interests or personal relationships that could have appeared to influence the work reported in this paper.

## References

[1] Ebraheim N.A. (1996). Calcaneocuboid joint involvement in calcaneal fractures. Foot Ankle Int..

[2] Carr J.B., Hammilton J.J., Bear L.S. (1989). Experimental intra-articular calcaneal fractures: anatomic basis for a new classification. Foot Ankle.

[3] Hctchinson F., Huebner M.K. (1994). Treatment of os calcis fractures by open reduction and internal fixation. Foot Ankle Int..

[4] Stephanson J.R. (1987). Treatment of displaced intra-articular fractures of the calcaneus using medial and lateral approaches, internal fixation, and early motion. JBJS.

[5] Gallino R.M., Gray A.C., Buckley R.E. (2009). The outcome of displaced intra-articular calcaneal fractures that involve the calcaneocuboid joint. Injury.

